# The evolution of the medical workforce in Cape Verde since independence in 1975

**DOI:** 10.1186/s12960-017-0180-9

**Published:** 2017-01-18

**Authors:** A. P. Delgado, A. C. Tolentino, P. Ferrinho

**Affiliations:** 1 Global Health and Tropical Medicine, GHTM, WHO Collaborating Centre on Health Workforce Policy and Planning, Instituto de Higiene e Medicina Tropical, IHMT, Universidade Nova de Lisboa, UNL, Rua da Junqueira 100, 1349-008 Lisboa, Portugal; 2 Fundação Amílcar Cabral, Praia, Cape Verde

**Keywords:** Cape Verde, Cuban Health Diplomacy, Feminization, Medical education, Workforce development

## Abstract

**Background:**

Cape Verdean doctors have always graduated abroad. The first experience of pre-graduate medical education in Cape Verde begun in October 2015. Counting how many doctors Cape Verde has, knowing who they are, and knowing how they are distributed are very important to help fine-tune the medical training. The aim of this study is to analyze the evolution of the medical workforce in Cape Verde to support medical education implementation.

**Methods:**

Secondary data on doctors, from July 1975 until December 2014, collected from the Ministry of Health, were entered into an SPSS 20 database and studied by a simple descriptive statistical analysis.

**Results:**

The database included data on 401 medical doctors. There was a predominance of females (*n* = 218; 54.4%). The overwhelming majority (*n* = 378; 94.3%) graduated from 5 of the 17 countries that contributed to the training of Cape Verdean doctors. All the islands of this archipelago country contributed to the 324 (80.8%) doctors born in the country.

Of the 272 doctors still active in December 2014, 119 (43.6%) were general practitioners and 153 (56.4%) had specialized in one of the 31 specialties.

The national ratio of doctors per 10 000 inhabitants was 5.25, but the reality varied significantly among islands.

About one third of the doctors (*n* = 86; 32%) were at the primary care level, 38 (14%) at the secondary care level, and 144 (52%) in central hospitals.

In 2053, all active physicians in 2014 will already be retired.

**Conclusions:**

This is a unique study of the evolution of the medical workforce of a country over 40 years, from the first day of independence.

The study illustrates the importance of international collaborations, particularly of Cuba, in sustaining the medical workforce in Cape Verde. It is an example of how this collaboration was used to equip the country with doctors in an increasingly more equitable distribution across all islands.

The study further illustrates the progressive feminization of the medical workforce.

The study clarifies the effort required from the emerging medical faculty to supply the national health system with the needed number of doctors.

## Background

The fact that the training of Cape Verdean doctors has always been made abroad, since before political independence in 1975, is determinant of the number and profile of basic and specialized training achieved. However, the achievements observed, although contributing to improve the cadre of competent professionals providing health services, have not been sufficient to meet the quantitative and especially the qualitative health needs of the resident population in the country.

The first experience of undergraduate medical education in the country has just begun in October 2015 with major support from the Faculty of Medicine of Coimbra (FMUC), Portugal, which seeks to replicate in the University of Cape Verde (Uni-CV) the model of medical education developed by the University of the Azores, Portugal, also supported by the FMUC [1].

To define policies and strategies and to manage and develop the professional capital in the health sector, it is necessary to know the people who work in it, how many there are, how they have evolved, and how they feel [2].

Hence, to count how many doctors Cape Verde has, know who they are, understand how they are distributed in the universe of its ten islands, and be acquainted with their rate of attrition are undoubtedly very important to help fine-tune the medical training just initiated. Therefore, the objective of this study is to analyze the evolution of the medical workforce in Cape Verde in the 40 years since independence, to support the implementation of medical education in the Uni-CV.

## Methods

For the characterization of the medical workforce in Cape Verde in the post-independence period, we used secondary data on doctors collected from the health workers’ administrative records in the Department of Human Resources in the Ministry of Health (MoH) from 5 July 1975, the date of the declaration of independence, until 31 December 2014. In some cases, particularly for the oldest and deceased, we sought additional information in personal files and with family members.

The data were entered into an SPSS 20 database. The variables included identification (name and number), date of birth, sex, date of initial medical training, date of recruitment to the Cape Verdean National Health Service (NHS), date and type of specialized training, date of re-entry after specialty training, place of current deployment, level of care placement (primary, secondary, and tertiary), and the situation on 31 December 2014 (active in the public sector, retired, on long leave, or deceased).

A simple descriptive statistical analysis was performed using SPSS 20 software for relative frequencies, median, mean, and standard deviation. As the study is a censitary study, means and frequencies are considered true values of the population; hence, analytical tests such as Student *t* tests and chi-square tests are not applied.

## Results

### Demographic characterization

**Fig. 1:**
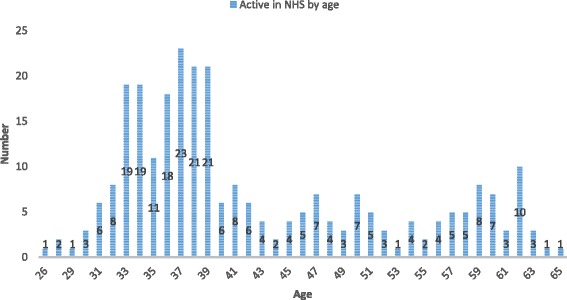
Age distribution of the active doctors

The database included data on 401 physicians. Among these, there was a predominance of females (*n* = 218, 54.4%) with an average age of 42.1 ± 13.7 years (for males, *n* = 183, the average age was 51.2 ± 15.1). Considering only doctors in active service in December 2014, the average age for women was 40.7 ± 9.5 and for men 45.5 ± 9.9. Most of them (*n* = 109, 67.3%) were females under 40 years, compared with an older male population (*n* = 44, 48.4% under 40 years), reflecting the feminization of medical workforce in the younger generations. This age distribution of the active workforce is represented in Fig. 1.

### Nationality

Most of the recruited doctors were born in Cape Verde (*n* = 324; 80.8%), while 77 others were born in 17 other countries, namely Cuba (*n* = 20; 5%), Guinea-Bissau (*n* = 17, 4.2%), Portugal (*n* = 11, 2.7%), Angola (*n* = 8, 2%), and Sao Tome and Principe (*n* = 4, 1%). Most foreign-born doctors adopted Cape Verdean nationality (many keeping dual nationality) mostly because of Cape Verdean ancestry (*n* = 31, 40.2%), or through marriage with Cape Verde indigenous spouses (*n* = 26, 33.5%) and they practice medicine as any other Cape Verde born national doctor.

### Contribution of each island to the medical workforce

All the islands contributed to the cadre of 324 doctors born in the country. By comparing the proportional weight of the resident population on each island with the percentage of doctors born there, it is possible to identify three patterns: S. Vicente, S. Antão, and S. Nicolau contributed to the national medical workforce above their share of the population; in Boa Vista, Sal, and Santiago, the inverse is observed, and the percentage of doctors born there is lower than their share of population; for Brava, Fogo, and Maio, these percentages are similar (Table 1).

**Table 1:** Relative contribution of each island to the medical workforce, 2014

Islands	Population^a^	% of total	Doctors born in^b^	% of total
S. Vicente	80 140	15.5	83	25.6
Santo Antão	41 192	7.9	43	13.3
S. Nicolau	12 511	2.4	14	4.3
Santiago	290 265	56.0	144	44.4
Boa Vista	13 376	2.6	4	1.2
Sal	32 208	6.2	7	2.2
Brava	5 760	1.1	5	1.5
Fogo	36 068	7.0	21	6.5
Maio	6 947	1.3	3	0.9
Cape Verde	518 467	100.0	324	100.0

^a^Source: INECV, Statistical Yearbook, 2015

^b^Source: Cape Verde MoH, Medical Database 2014

### Growth of the medical workforce

In July 1975, at the time of independence, Cape Verde remained with 13 doctors. The cumulative total increased to 401 doctors listed at the end of the study period, 31 December 2014.

**Fig. 2:**
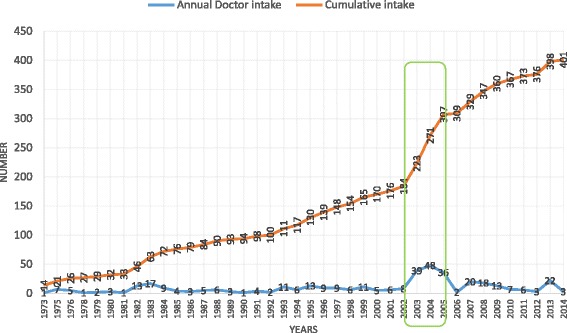
Doctor intake and cumulative total

Until 2003, there was a relatively slow growth of the medical workforce. From 2003 to 2005, there was a peak intake of 123 doctors who had been sent for training in Cuba. Four years later, 51 more doctors, who were sent to Cuba for training, returned with specialization in several areas (Fig. 2).

**Fig. 3:**
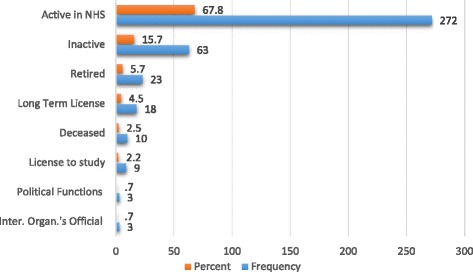
Doctor situation on 31 December 2014

It is also around this time that doctors start leaving the NHS in significant numbers for the most diverse reasons (Fig. 3): over the 40 years, there was a loss of 128 doctors and on 31 December 2014, only 272 of the 401 doctors (68%) remained active in the NHS.

### Feminization of the workforce

**Fig. 4:**
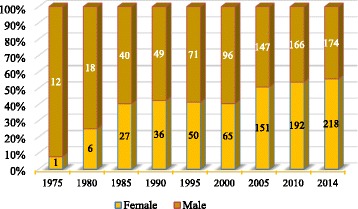
Feminization of the medical workforce

In 1975, among the 13 doctors in the country, only one was a woman. But, since then, the number of women grew rapidly, reversing the male-female ratio in 2005—in 2014, 218 females represented 54% of all doctors (Fig. 4). Considering only the practicing doctors, this proportion is even more accentuated—females represented 59% (*n* = 162) of the active workforce.

### Place of training

The 401 doctors who made up the national contingent graduated in 99 institutions of higher education in 17 countries. Five countries graduated 378 (94.3%) of these doctors. The remaining 23 received their training in 12 other countries.

Cuba appeared with the highest number of graduates (*n* = 189, 47.1%), almost half of the country’s doctors. This training was done in 16 higher education institutions, but with marked clustering in four: the Higher Institutes of Medical Sciences in Camaguey (*n* = 81, 42.9%), in Havana (*n* = 67, 35.4%), the Latin American School of Medicine (*n* = 17, 10.0%), and in Santiago de Cuba (*n* = 8, 4.2%).

Portugal contributed to the training of 78 (19.5%) doctors in the four oldest Portuguese medical schools: Lisbon (*n* = 45, 57.7%), New Lisbon (*n* = 17, 21.8%), Coimbra (*n* = 13, 16.7%), and Oporto (*n* = 3, 3.8%).

Brazil comes third, training 76 (19.0%) doctors in 30 different universities, but without significant clustering.

The former Union of Soviet Socialist Republics (USSR) comes fourth having trained 36 (9.0%) Cape Verdean doctors, especially in the early years after independence, in the 1980s, once again with a great dispersion of training sites.

**Fig. 5:**
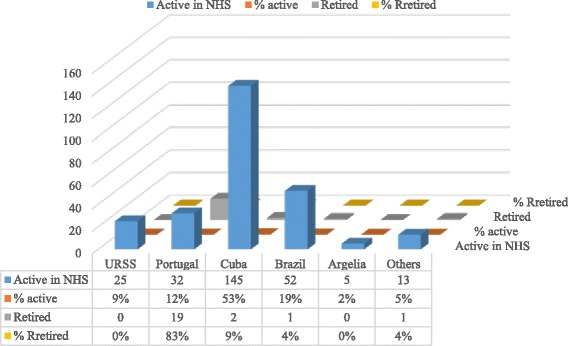
Place of initial medical training

The clustering of places of initial medical training changed over time. While older retired doctors (*n* = 23) graduated mostly in Portugal (*n* = 19, 83%), younger doctors, trained after independence and still active in the NHS in 2014 (*n* = 272), graduated mostly in Latin American countries—Cuba (147, 54%) and Brazil (52, 19%)—and also in Portugal (*n* = 32, 12%), USSR (*n* = 25, 9%), and Algeria (*n* = 5, 2%) and the remaining 5% (*n* = 13) in other countries (Fig. 5).

**Fig. 6:**
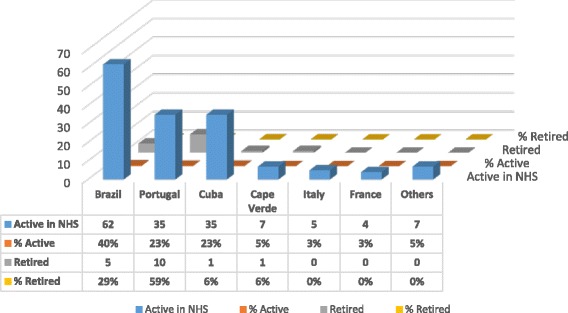
Place of specialist training

Analyzing the place of specialist training (Fig. 6), the same clustering pattern was observed. Retired doctors practiced mostly as general practitioners, but those that specialized (*n* = 17) did so mostly in Portugal (*n* = 10, 58.8%), followed by Brazil (*n* = 5, 29.4%), Cuba (*n* = 1, 6%) and Cape Verde (*n* = 1, 6%). Active specialist doctors (*n* = 155) were trained mostly in Latin America (Brazil with 40% (*n* = 62), Cuba with 23% (*n* = 35), also in Portugal with 23% (*n* = 35), and Cape Verde with 5% (*n* = 7)).

**Fig. 7:**
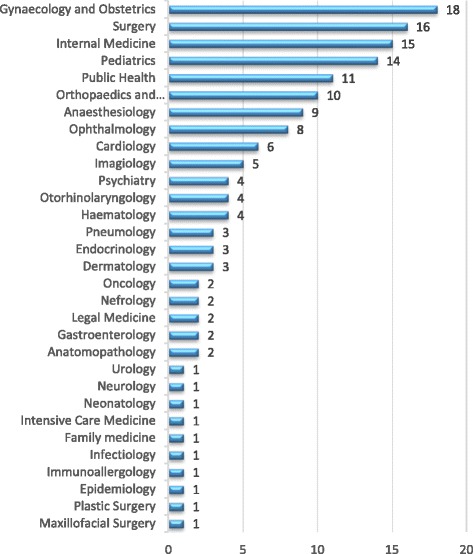
Doctors’ distribution by specialties

Of the 272 active doctors, less than half (*n* = 119, 43.6%) consisted of general practitioners without specialization. The 153 specialist doctors were concentrated into five “basic” specialties (general surgery, anesthesiology, internal medicine, pediatrics, obstetrics and gynecology) with 72 doctors (47%). The other 26 clinical specialties were dispersed by 69 doctors (45%), with the particularity of ten of them having only one medical specialist and five others two each. The three public health specialties (public health, epidemiology, and family medicine) held only 13 doctors (8%) (Fig. 7).

### Distribution of doctors by islands

The 2003–2005 intake of doctors changed substantially their distribution, allowing the placement of at least two doctors in each island and in less populated isolated municipalities.

On 31 December 2014, nationally, there were 5.25 doctors per 10 000 inhabitants, but the reality varied significantly among islands. The largest island, Santiago, shared the average (5.20), while the second most populated island, S. Vicente, was well above the average (9.23) and all the other islands were below the national average (Table 2).

**Table 2:** National distribution of doctors per 10 000 inhabitants, 2014

Islands	Population 2014^a^	Doctors^b, c^	Doctors by 10 000 population
S. Vicente	80 140	74	9.23
Santiago	290 265	151	5.20
Brava	5 760	2	3.47
S. Nicolau	12 511	4	3.20
Sal	32 208	9	2.79
Fogo	36 068	10	2.77
Boa Vista	13 376	3	2.24
Santo Antão	41 192	12	2.91
Maio	6 947	1	1.44
Cape Verde	518 467	272	5.25

^a^Source: Statistical Yearbook 2015 INECV

^b^Six doctors were placed at national level (MoH) in 2014

^c^Source: MoH, Medical Database 2014

### Doctors’ distribution by levels of care and geography

Of the total active medical workforce, 86 (32%) physicians were in the primary health care level, mostly general practitioners placed in health centers (n = 73, 84.9%). The lowest rate per 10,000 inhabitants was in Sal and the highest in Brava (Table 3); 38 doctors (14%) were at the secondary health care level in regional hospitals (RH) and the largest health centers, including 17 general practitioners, 14 doctors with basic specialties, and seven with other specialties, with the lowest rates observed for the Region of Praia/S. Domingos and the highest for S. Vicente/S. Nicolau (Table 4); and 144 (52%) were in central hospitals (CH) to provide tertiary and secondary health care, mostly with other (*n* = 66) or basic specialties (*n* = 47), including 28 general practitioners (Table 5).

**Table 3:** Doctors’ distribution at primary care level and island, 2014

Islands	Doctors^a^	Population^b^	Doctors per 10 000 population
Brava	2	5 760	3.47
S. Nicolau	4	12 511	3.20
S. Vicente	18	80 140	2.25
Boa Vista	3	13 376	2.24
Santo Antão	9	41 192	2.18
Fogo	6	36 068	1.66
Santiago	45	290 442	1.55
Maio	1	6 947	1.44
Sal	1	32 208	0.31
Cape Verde	89	518 644	1.72

^a^Source: Cape Verde MoH, Medical Database 2014

^b^Source: INECV, Statistical Yearbook 2015

**Table 4:** Doctors’ distribution at secondary care and health region, 2014

Health regions/HR	Doctors^d^	Population^e^	Doctors per 10 000 population
HRFB/Fogo e Brava	7	41 828	1.67
HRSA/Santo Antão	7	41 192	1.70
HRBS/Boa Vista e Sal^a^	8	45 584	1.76
HRMi^b^/S. Vicente e S. Nicolau^a^	18	92 651	1.94
HRSN/Santiago Norte	23	120 252	1.91
HRSS^b^/Santiago Sul^a, c^	28	177 137	1.58
Cape Verde	91	518 644	1.75

^a^HR did not formally created

^b^Estimated number of doctors

^c^Praia, S. Domingos, Cidade Velha, and Maio

^d^Source: Cape Verde MoH, Medical Database 2014

^e^INECV, Statistical Yearbook 2015

**Table 5:** Doctors’ distribution at tertiary care, national level, 2014

National Hospitals	Doctors^a^	Population^b^	Doctors per 10 000 population
Hosp. Central Agostinho Neto	57	339 217	1.68
Hosp. Central Baptista de Sousa	38	179 427	2.12
Cape Verde	95	518 644	1.83

^a^Source: Cape Verde MoH, Medical Database 2014

^b^INECV, Statistical Yearbook 2015

### Predicting the retirement of doctors

The process of retirement from Cape Verdean public service has followed one of two criteria: one based on length of service, with a 34-year minimum, which can be activated after the age of 60 years and another criterion is the age for compulsory retirement at 65 years, independent of years of service.

**Fig. 8:**
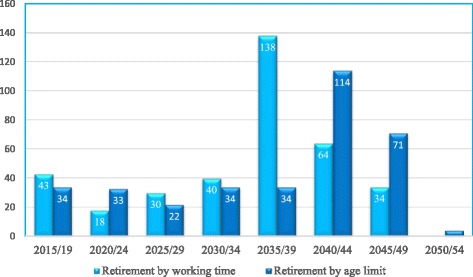
Predicting retirement of doctors by a 5-year period

An analysis of the distribution of doctors per their current age allows us to time the retirement of doctors in active service. At the time of the study, doctors under 40 years were the majority (*n* = 156, 57.3%), with an expected working life of 20 to 25 years before retirement, while 55 (20.3%) had between 40 and 50 years of age, 44 (16.2%) between 50 and 60 years, and 17 (6.2%) over 60 years. This suggests that all doctors in active duty on 31 December 2014 will be retired by 2053 whatever the criteria applied. This implies that:

- ○ Thirty-four to forty-three doctors will retire during the period 2015–2019. During this period, the local medical training program will not have yet graduated any student.
- ○ Eighteen to thirty-three doctors will retire from 2020 to 2024. The local medical program will have the first graduation in 2021, with an expected output of 80 doctors for the period.
- ○ From 2025 to 2029, 22–30 retirements are expected.
- ○ From 2030 to 2034, the retirement level begins to rise to 34–40, taking one or the other of the criteria.
- ○ From 2035 to 2039, the pressure for retirement will be at its highest, with 34 doctors who must leave because they will have reached the age limit and 138 who will complete 34 years of service and 60 years of age and may request retirement.
- ○ From 2040 to 2044, the retirement of doctors will be high and may reach 114 by the age limit criterion or 34 by the criteria of 34 years of service and 60 years of age.
- ○ By 2045–2049, the retirement expectations remain high around 70 by the criterion of age limit plus 34 years of service (Fig. 8).

It is expected that the medical training program will be able to meet the local needs for doctors for each of the periods from 2021 onwards.

## Discussion

The numbers presented reflect the major success of Cape Verde in mobilizing national doctors to equip its health system. This is even more astounding if we consider that immediately after independence, in July 1975, the country remained with 13 doctors, half of the medical workforce of 25 doctors described during colonial times, in 1953 [3].

Cape Verde’s health system has evolved from an undifferentiated national health system to an increasingly more regionalized one, drawing on the natural conditions (insularity, small population clusters) and mustering on the relative scarcity of human, material, and financial resources [4].

### Training medical doctors

At the time of independence, Cape Verde met the immediate medical needs of the population with the support of a well-thought international health cooperation program, particularly with Cuba. Cuba sent “brigades” of health workers, allowing Cape Verde to extend its health services’ coverage to all islands, strengthening local health activities. This dependence has been much reduced by the policy sending medical students to train abroad until conditions made it possible to start local medical training.

The growth of the national workforce has been sustained and has been achieved with the support of undergraduate training in 17 countries, mostly in other Portuguese-speaking countries (Portugal or Brazil) and Cuba. The choice of Cuba is best understood in the context of Cuba’s willingness to receive the Cape Verdean students, as part of its medical aid activities [5, 6] assuring these students of the learning conditions and subsistence support needed, based on agreements signed between both governments.

Like for other countries, the stress of accommodating professionals coming from a great diversity of training institutions in many countries was one of the main motivating factors for the development of local medical training [7]. This will reduce the need to send students abroad for training, probably requiring a novel form of future support in terms of visiting professorships to support the local medical school to overcome the shortage of trained teachers [8] (Delgado, Martins, Ferrinho: The experience of medical training and student’s expectations at Uni-CV, submitted).

Despite the recognition of this adaptation stress for returning medical students, unlike other countries that also benefited from Cuban cooperation to train their medical personnel [9], Cape Verdean authorities never developed a successful reintegration program to support returning doctors to overcome the differences of culture and clinical contexts between Cuba and Cape Verde. This reintegration support, although intended, was mostly left to the peers welcoming newcomers to the service benefiting from the new recruitment.

Our numbers do not reveal the inefficiency inherent in the system of sending Cape Verdean to train abroad, as they do not reflect those that did not return to Cape Verde, as such losses have not been documented.

The same inefficiency has been observed for specialist training. It is encouraging to note that about 5% of the medical specialists have been trained in Cape Verde. This figure reflects two experiences that convey the capacity to develop specialist training in Cape Verde but still lacking the elements to make this training sustainable without outside support. The first experience in local specialist training was developed in the 1990s, with the support of the FMUC, and trained five specialists in obstetrics and gynecology. The second experience, with the support of IHMT-UNL, trained six public health specialists. Both experiences implied, for some of the doctors trained, short internships in Portugal (6–12 months). Local capacity for specialist training is important to improve care and to retain doctors in the country.

The numbers of registered doctors in relation to the general population indicate that the country is today substantially better supplied with doctors than any other sub-Saharan African country, except for South Africa [10–12], but grossly undersupplied when compared with developed countries [13, 14]. This may account for the fact that Cape Verde has some of the best health status indicators for African countries [15].

### Deployment of doctors

The increased number of returning students as doctors, in a country where the government has been and remains their major employer, has allowed the placement of at least two national doctors in each island and at least one in less populated and isolated municipalities.

Despite the increase in the number of doctors, there still remain large deployment inequalities among different islands and health regions in the country [16, 17].

The irregular distribution by islands may, in part, be explained by internal migration of the population of S. Vicente, S. Antão, S. Nicolau, and Brava, where the population has been growing at a very small rate or decreasing [18], to the more touristic islands of Sal, Boa Vista, and Santiago (with the country’s capital) which have attracted people in the mirage of better jobs.

It may also be explained by the concentration of health care facilities (particularly secondary and tertiary care level facilities) in some islands, illustrating that in island states like Cape Verde, the need to guarantee a minimum package of care in every island, independent of size of population, defeat attempts to follow rational economies of scale [19], which may explain some high resource concentrations observed for islands like Brava.

The disparities are even more significant when considering the different medical specialties. These disparities point to the need to take geographical factors into consideration as part of the criteria of selection of future medical students.

### Characterization of the medical workforce

The medical workforce is mostly undifferentiated, with only 56% having a medical specialty (2.9 per 10 000 population). Nevertheless, when compared to other African countries, Cape Verde is better served by medical specialists [2, 20] but, again, with ratios much lower than developed countries [3].

Medical training should consider the feminization of the medical workforce [21]. Data from elsewhere suggest that feminization may bring changes in several dimensions of medical practice [22, 23]. The marked feminization we describe was confirmed in another independent study comparing the medical workforce in the capital cities of Cape Verde (Praia), Mozambique (Maputo), and Guinea-Bissau (Bissau). Feminization was highest in Praia (56.4%) and lowest in Bissau (28.1%). Overall, in this study, female doctors were younger than males in the three locations but less likely to hold a specialty. They also worked shorter hours per week—probably to accommodate traditional domestic roles which, in Cape Verde, still weigh heavily on women who have to struggle with a relative lack of child care facilities—with hours spent in the private sector accounting for much of the difference [24–26].

Besides geographical considerations, yearly intakes for the local medical course should also consider the expected rate of retirement. By 2053, all active physicians enrolled in 2014 will be retired, and in a 32-year period, (2021–2053) the medical training program in Cape Verde will have graduated about 650 doctors, more than correcting the losses observed. This may not be enough, as the output of the medical training program should take into account other considerations, like population growth, brain drain, and migration, which are not within the scope of this paper.

## Conclusions

This is a unique study of the evolution of the medical workforce of a country over 40 years from the first day of independence.

The study illustrates the importance of international collaborations, particularly with Cuba’s health diplomacy, in sustaining the medical workforce in Cape Verde, while Cape Verdean students were sent abroad for training as medical doctors. It is an example of how this collaboration was used to equip the country with more doctors that could then be distributed to all islands and all levels of care, progressively replacing foreign doctors with national ones upon their return from training abroad.

The study clarifies the effort required from the emerging medical faculty to supply the national health system with the needed number of doctors and to support the process of medical specialization.

The lack of local post-graduate medical training, a medical workforce still mostly undifferentiated, the heavy feminization, the need to take geographical factors into consideration as part of the criteria of selection of medical students to correct for large deployment inequalities among different islands and Health Regions in the country, and the attrition rate of doctors due to retirement should be considered to fine-tune the medical training just initiated in the country.

## Abbreviations

CH: Central hospitals
FMUC: Faculty of Medicine of Coimbra University
HR: Health region
HRBS: Health region of Boa Vista and Sal islands
HRFB: Health region of Fogo and Brava islands
HRMi: Health region of Mindelo (S. Vicente and S. Nicolao islands)
HRSA: Health region of Santo Antão island
HRSN: Health region of Santiago Norte (the north of Santiago island)
HRSS: Health region of Santiago Sul (The south of the island of Santiago and Maio Island)
IHMT-UNL: Institute of Hygiene and Tropical Medicine-New University of Lisbon
MoH: Ministry of Health
NHS: National Health Service
RH: Regional hospitals
Uni-CV: University of Cape Verde
USSR: Union of Soviet Socialist Republics

## References

[1] Regateiro FJ (2014). Curso de Medicina: Enquadramento e Plano de Estudos, versão 3.0. Universidade de Cabo Verde/Universidade de Coimbra.

[2] Martins J, Biscaia A, Conceição C, Fronteira I, Hipólito F, Carrolo M, Ferrinho P (2003). Caracterização dos profissionais de saúde em Portugal Parte I—Quantos somos e quem somos. Revista Portuguesa Clinica Geral.

[3] Cabral E (1958). Os Serviços de Saúde da Província de Cabo Verde. Anais do IMT.

[4] Scheffler RM, Liu JX, Kinfu Y, Dal Poz MR (2008). Forecasting the global shortage of physicians: an economic- and needs-based approach. Bull World Health Organ.

[5] Huish R, Kirk JM (2007). Cuban medical internationalism and the development of the Latin American School of Medicine. Lat. Am. Perspect..

[6] Kirk JM, Erisman HM (2009). Cuban medical internationalism.

[7] Anyangwe S, Mtonga C (2007). Inequities in the global health workforce: the greatest impediment to health in sub-Saharan Africa. Int J Environ Res Public Health.

[8] Fresta MJ, Ferreira MA, Delgado AP, Sambo MR, Torgal J, Sidat M, Ferrinho P (2016). Estabelecimento de uma rede estruturante da cooperação em educação médica, no âmbito do PECS-CPLP—towards a PECS-CPLP network for medical education. An Inst Hig Med Trop.

[9] Gasiorek J, Van de Poel K (2014). ‘We feel stupid and we shouldn’t.’ Towards developing a communication support system for Cuban-trained medical students. Per Linguam: a Journal of Language Learning = Per Linguam: Tydskrif vir Taalaanleer.

[10] World Health Organization.http://gamapserver.who.int/gho/interactive_charts/health_workforce/PhysiciansDensity_Total/atlas.html.

[11] Kinfu Y, Dal Poz MR, Mercer H, Evans DB (2009). The health worker shortage in Africa: are enough physicians and nurses being trained?. Bull World Health Organ.

[12] Breier M (2008). Research commissioned by Department of Labour South Africa: the shortage of medical doctors in South Africa.

[13] OECD (2016). Doctors (indicator).

[14] Delgado APC (2009). Políticas de Saúde em Cabo Verde na década de 1980-1990: Experiência de Construção de um Sistema Nacional de Saúde.

[15] World Health Organization. Health Situation Analysis in the African Region: Atlas of Health Statistics. Africa Brazzaville/Republic of Congo: WHO; 2011.

[16] Biscaia A, Conceição C, Martins J, Ferrinho P (2003). Política e gestão dos recursos humanos na Saúde em Portugal–controvérsias. Revista Portuguesa de Clínica Geral.

[17] Russo G, Ferrinho P, de Sousa B, Conceição C (2012). What influences national and foreign physicians’ geographic distribution? An analysis of medical doctors’ residence location in Portugal. Hum. Resour. Health.

[18] Cabo Verde Instituto Nacional de Estatísticas (2015). Anuário Estatístico.

[19] Walsh K (2014). Economies of scale in medical education? Or diseconomies of scale?. East Mediterr Health J.

[20] Fronteira I, Sidat M, Fresta M, do Rosário Sambo M, Belo C, Kahuli C, Rodrigues MA, Ferrinho P. The rise of medical training in Portuguese speaking African countries. Human resources for health. 2014;12(1):1. 10.1186/1478-4491-12-63 PMC4232702 25367224

[21] Mundial B. Relatório sobre o Desenvolvimento Mundial-2012. Igualdade de Gênero e Desenvolvimento. VisãoGeral. Washington, DC: Banco Mundial; 2012.

[22] do Céu Soares Machado M. A feminização da medicina. Análise social. 2003;38(166):127–37.

[23] Eisenberg C (1989). Medicine is no longer a man’s profession. N. Engl. J. Med..

[24] Russo G, Gonçalves L, Craveiro I, Dussault G (2015). Feminization of the medical workforce in low-income settings; findings from surveys in three African capital cities. Hum. Resour. Health.

[25] Hedden L, Barer ML, Cardiff K, McGrail KM, Law MR, Bourgeault IL (2014). The implications of the feminization of the primary care physician workforce on service supply: a systematic review. Hum. Resour. Health.

[26] Contandriopoulos AP, Fournier MA (2007). Féminisation de la profession médicale et transformation de la pratique au Québec. Groupe de recherche interdisciplinaire en santé, Faculté de médecine, Université de Montréal.

